# Elevated SCN11A concentrations associated with lower serum lipid levels in patients with major depressive disorder

**DOI:** 10.1038/s41398-024-02916-w

**Published:** 2024-05-11

**Authors:** Ke Xu, Shuang Zhao, Yi Ren, Qi Zhong, Jinzhou Feng, Dianji Tu, Wentao Wu, Jiaolin Wang, Jianjun Chen, Peng Xie

**Affiliations:** 1 Department of Neurology, The First Affiliated Hospital of Chongqing Medical University, Chongqing, China; 2 National Health Commission Key Laboratory of Diagnosis and Treatment on Brain Functional Diseases, The First Affiliated Hospital of Chongqing Medical University, Chongqing, China; 3 Department of Infectious Diseases, Key Laboratory of Molecular Biology for Infectious Diseases, Ministry of Education, Institute for Viral Hepatitis, The Second Affiliated Hospital of Chongqing Medical University, Chongqing, China; 4 Lab of Stem Cell and Tissue Engineering, Department of Histology and Embryology, Chongqing Medical University, Chongqing, China; 5 Institute of Life Sciences, Chongqing Medical University, Chongqing, China; 6 Department of Clinical Laboratory, Xinqiao Hospital, Third Military Medical University (Army Medical University), Chongqing, China

**Keywords:** Depression, Diagnostic markers

## Abstract

The pathogenesis of major depressive disorder (MDD) involves lipid metabolism. Our earlier research also revealed that MDD patients had much lower total cholesterol (TC) concentrations than healthy controls (HCs). However, it is still unclear why TC decreased in MDD. Here, based on the Ingenuity Knowledge Base’s ingenuity pathway analysis, we found that sodium voltage-gated channel alpha subunit 11A (SCN11A) might serve as a link between low lipid levels and MDD. We analyzed the TC levels and used ELISA kits to measure the levels of SCN11A in the serum from 139 MDD patients, and 65 HCs to confirm this theory and explore the potential involvement of SCN11A in MDD. The findings revealed that TC levels were considerably lower and SCN11A levels were remarkably increased in MDD patients than those in HCs, while they were significantly reversed in drug-treatment MDD patients than in drug-naïve MDD patients. There was no significant difference in SCN11A levels among MDD patients who used single or multiple antidepressants, and selective serotonin reuptake inhibitors or other antidepressants. Pearson correlation analysis showed that the levels of TC and SCN11A were linked with the Hamilton Depression Rating Scales score. A substantial association was also found between TC and SCN11A. Moreover, a discriminative model made up of SCN11A was discovered, which produced an area under a curve of 0.9571 in the training set and 0.9357 in the testing set. Taken together, our findings indicated that SCN11A may serve as a link between low lipid levels and MDD, and showed promise as a candidate biomarker for MDD.

## Introduction

Major depressive disorder (MDD) is a common mental disorder with high mortality and disability [1–4]. Persons with unremitted depression are more likely to commit suicide [5, 6]. However, only 30-40% remit with antidepressant treatment leaving nearly 60-70% of patients who do not optimally respond [7]. Increased knowledge suggested that lipid abnormalities play a significant part in the pathophysiology of MDD [8–11], which may open up a new avenue for research into the underlying mechanism of MDD.

Similar to other studies [12, 13], our earlier research [14] found that though there was no significant difference in high-density lipoprotein cholesterol (HDL-C) and triglyceride (TG) between the MDD patients and healthy controls (HCs), the total cholesterol (TC) and LDL-C levels were much lower in MDD compared to HCs. Meanwhile, the TC levels were positively correlated with LDL-C levels, and the reduction of TC was connected with the severity of MDD [9], suggesting that low lipid levels may play a role in the pathogenesis of MDD. In addition, the absence of a specific molecular basis for diagnosis in psychiatry at this time prevents the creation of diagnostic tools, and diagnosis in MDD is still reliant on symptomatology. In recent years, peripheral tissues, such as blood, are gradually being used to study psychiatric illnesses [15–18], and some molecular mediators found in these studies may be used as possible biomarkers [19–22]. Thus, serum investigations are necessary to understand how they function in MDD and if they have potential as candidate biomarkers for the diagnosis of MDD. It also should not ignore the molecule that is associated with low lipid levels.

As a stressor, chronic pain is one of the key elements in determining depression [23–25], and up to 85% of chronic pain patients have severe depression [26, 27]. The role of sodium channels in chronic pain disorders has been well recognized [28, 29]. Na_v_1.9, which is encoded by sodium voltage-gated channel alpha subunit 11A (SCN11A), is one of the several sodium channel subtypes and is particularly relevant in the context of nociception. It was also shown that Na_v_1.9 is a significant modulator of peripheral pain hypersensitivity. SCN11A has thus been proposed as a desirable target for potent pain treatments [30]. In addition, cholesterol has been linked to Na_v_1.9 channel-mediated inflammatory pain [31]. These studies suggested that lipid metabolism may be associated with SCN11A disruption. Nevertheless, nothing is currently known regarding SCN11A expression and function in MDD patients. Therefore, more research on this crucial molecule in MDD is needed since the findings could reveal a useful network between serum lipids, SCN11A, and MDD.

In this study, we investigated the potential involvement of SCN11A in MDD by examining the blood levels of TC and SCN11A in MDD patients. Meanwhile, the correlations between SCN11A and both clinical traits and serum TC levels were also explored. Moreover, the potential for SCN11A to be employed as a biomarker for MDD and therapy response was evaluated.

## Methods and materials

### Subject recruitments

The Chongqing Medical University’s local ethics committee approved this study (No.2017013). Before taking blood samples, each participant gave their written informed permission. All the patients enrolled in this study were from the same cohort in our other recently published study [14]. In total, 139 MDD patients and 65 HCs were included. According to the Diagnostic and Statistical Manual of Mental Disorders-version IV criteria and the International Statistical Classification of Diseases and Related Health Problems criteria, 11^th^ revision, the diagnosis of MDD was validated by two qualified psychiatrists. The 17-item Hamilton Depression Rating Scale (HDRS) was used to assess the severity of depression in MDD patients [32, 33]. All MDD patients enrolled from the First Affiliated Hospital of Chongqing Medical University’s Department of Psychiatry. As antidepressants may have an impact on blood lipids, patients with MDD were split into two groups: those who had never taken drugs before (drug-naïve MDD, DN-MDD; *n* = 73) and those who had only received antidepressant treatment before (drug-treatment MDD, DT-MDD; *n* = 66). During the same period, HCs were recruited from the Medical Examination Center, First Affiliated Hospital of Chongqing Medical University. All patients and controls recruited in this study were Chinese Han population. We excluded individuals with additional physical issues and HCs with a history of neurological, DSM-IV Axis I/II, or other medical illnesses to prevent the impact of other variables. Meanwhile, participants who had used statins, cortisone, interferon, or fatty acid supplements in the three months prior were also eliminated from all groups. Age, sex, and body mass index (BMI) were matched between HCs and MDD patients.

### Clinical data collection

From the patient discharge letters, clinical information was taken, including the duration of illness, and detailed data on antidepressant usage was collected in the DT-MDD subgroup. We also gathered data from patient’s information on their marital status (single/married/divorced/widowed), education levels (low/middle/high), employment status (unemployed/others not working/working/in training/retired/housewife/-man), drinking and smoking habits (never/moderate/heavy).

### Ingenuity Knowledge Base’s ingenuity pathway analysis (IPA)

The IPA is a precise online biomedical analytic tool that aids in the prediction of current molecular interaction networks and the understanding of their features [34]. Here, based on prior research [35, 36], the IPA database was utilized to examine the possible network of correlations between cholesterol and MDD. We searched for possible networks between cholesterol and MDD using the ‘Grow’ and ‘Path Explorer’ tools and two keywords (cholesterol and MDD).

### Serum TC level analyses

Blood for TC level analyses was sampled at 7:00 AM after approximately 10–12 hours of overnight fasting. The samples were transported to the Center for Clinical Molecular Medical Detection, First Affiliated Hospital of Chongqing Medical University within one hour. Levels of TC were determined on the COBAS C8000 Modular Analyzer (Roche Diagnostics, Mannheim, Germany) according to routine laboratory methods. The normal value range for TC was 2.8–5.2 mM.

### Enzyme-linked immunosorbent assay (ELISA) analyze

Each participant’s peripheral whole-blood samples were taken by venous puncture. According to our earlier works [14, 32, 36, 37], serum was extracted by centrifuging it at room temperature for 15 minutes at a speed of 3000 g, then aliquoted, and stored at –80 °C until use. Two blind researchers used commercially available high-sensitivity ELISA kits from MEIMIAN (Jiangsu, China) to measure the total amount of SCN11A. All ELISA kits were used following the manufacturer’s instructions. The reference standard was used on ELISA plate to make a standard curve. Six standards (200, 100, 50, 25, 12.5, and 0 pg/mL) with varying concentrations ranging from 200 pg/mL to 0 pg/mL were prepared. The wells were filled with fifty microliters of standard or diluted serum samples (1: 4). After sealing the plate, it was incubated on a 400 rpm plate shaker for 30 min at 37 °C. Following five 30 s washes with 350 μL of wash buffer (1×), each well was then incubated with 50 μL of streptavidin-HRP on a shaker for 1 hour at 37 °C. After the final wash, each well received 100 μL of 3, 3’, 5, 5’-tetramethylbenzidine (TMB) development solution, which was then incubated for 10 min at 37 °C in the dark. The color changed from blue to yellow when 50 μL of stop solution was added and allowed to sit at room temperature for one minute on a plate shaker with a 400 rpm setting. Using an ELISA reader (Bio-Rad, Hercules, CA, USA), absorbance was measured at 450 nm. The defined standard curve was used to represent the data in pg/mL. The SCN11A assay’s detection limit was 4 pg/mL. The duration of sample collection and storage was the same for all groups.

### Statistical analysis

Statistical Package (version 20; IBM Corp., Armonk, NY, USA) was used to conduct statistical analyses. The results were shown as mean ± standard error of the mean (SEM). When comparing continuous demographic and clinical data between groups, independent sample *t*-tests, Mann–Whitney *U* test, Kruskal–Wallis test, or ANOVA followed by post hoc comparison with the Bonferroni test were used depending on whether the data fit the normal distribution between two or more groups. Bonferroni test, also known as ‘Bonferroni correction’ or ‘Bonferroni adjustment’, is an adjustment to the significance level used to evaluate the statistical significance of an individual comparison. Levene’s test was performed to determine the variances between MDD and HCs; if the variances were not comparable between the two groups, the adjusted *p*-value was applied. Using the Chi-square test, categorical variables were compared. The correlation between the two variables was described using the Pearson correlation coefficient. Considering the importance of independent samples in identifying potential biomarkers, the included samples were randomly divided into a training set (44 HCs and 92 MDD (50 DN-MDD, and 42 DT-MDD)) and a testing set (21 HCs and 47 MDD (23 DN-MDD, and 24 DT-MDD)). The logistic regression analysis was utilized to find a possible discriminative model for MDD, and receiver operating characteristic (ROC) curve analysis was performed to measure its diagnostic performance in distinguishing MDD patients from HCs in both training and testing sets. The value of the area under the ROC curve (AUC) served as an evaluation metric for diagnostic performance, and AUC = 0.90–1.00 was defined as ‘excellent’ [38–40]. *p* < 0.05 was defined as significant.

## Results

### Clinical and sociodemographic information

Table 1 displays that there were 65 HCs and 139 MDD patients (73 DN-MDD and 66 DT-MDD) overall. There was no significant difference in sex (*p* = 0.426), age (*p* = 0.629), or BMI (*p* = 0.117) distributions among the three groups. The DN-MDD and DT-MDD groups showed significantly higher HDRS scores (both *p* = 0.000) than the HCs group. Moreover, the DT-MDD group had significantly lower HDRS scores (*p* = 3.29E-4) than the DN-MDD group. Additionally, as shown in Table S1, the majority of patients were married, educated, employed, and never drank or smoked. Selective serotonin reuptake inhibitors (SSRIs; including escitalopram (21.43%), fluoxetine (8.93%), and sertraline (7.14%)) and selective serotonin-norepinephrine reuptake inhibitor (SNRI; including Venlafaxine (13.39%)) constituted the bulk of the antidepressant drugs taken by DT-MDD patients.

**Table 1:** Clinical details of recruited subjects in the study.

Characteristics	HCs	DN-MDD	DT-MDD	*p*-value
Sample size (*n*)	65	73	66	-
Sex (male/female)	27/38	37/36	27/39	0.426^a^
Age (years)				
Range	19–60	18–60	18–66	-
Mean ± SEM	36.38 ± 1.23	36.14 ± 1.48	38.56 ± 1.78	0.629^b^
BMI	22.48 ± 0.25	21.64 ± 0.24	22.20 ± 0.17	0.117^b^
HDRS (mean ± SEM)	3.23 ± 0.38	32.53 ± 1.02	27.48 ± 0.75	1.10E-30^c,d^
Duration of illness (months)				
Range	-	1–120	1–180	-
Mean ± SEM	-	28.43 ± 4.33	53.74 ± 5.67	-

Continuous variables are expressed as mean ± standard error of the mean (SEM).

*BMI* body mass index, *HCs* healthy controls, *HDRS* Hamilton depression rating scale, *MDD* major depressive disorder.

^a^Analyzed by the Chi-square test.

^b^Analyzed by Kruskal–Wallis *H*-test.

^c^Analyzed by one-way analysis of variance.

^d^Tamhane’s T2 post hoc test was conducted to determine which groups differed significantly: DN-MDD vs. DT-MDD (*p* = 3.29E-4), DN-MDD vs. HCs (*p* = 0.000), DT-MDD vs. HCs (*p* = 0.000).

### IPA analysis

**Fig. 1:**
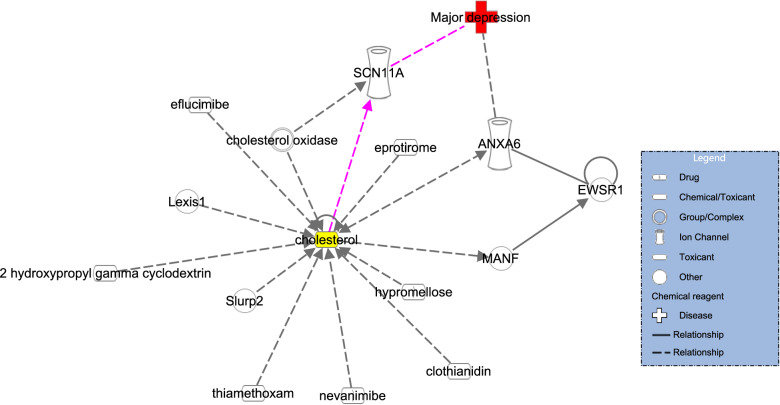
Potential networks between cholesterol, SCN11A, and major depressive disorder. According to the Ingenuity Pathway Analysis database, SCN11A may serve as the link between cholesterol and major depressive disorder. *ANXA6*, annexin A6; *EWSR1*, ewing sarcoma breakpoint region 1; *Lexis1*, lipid responsive LXR-induced inhibitor of cholesterol synthesis 1; *MANF*, mesencephalic astrocyte-derived neurotrophic factor; *SCN11A*, sodium voltage-gated channel alpha subunit 11A; *SLURP2*, secreted mammalian Ly6/urokinase plasminogen activator receptor-related protein (SLURP)-2.

According to the analysis of the discovered molecular network, cholesterol might be involved in the pathogenesis of MDD by regulating the SCN11A (Fig. 1).

### Serum concentrations of TC and SCN11A in MDD patients

**Fig. 2:**
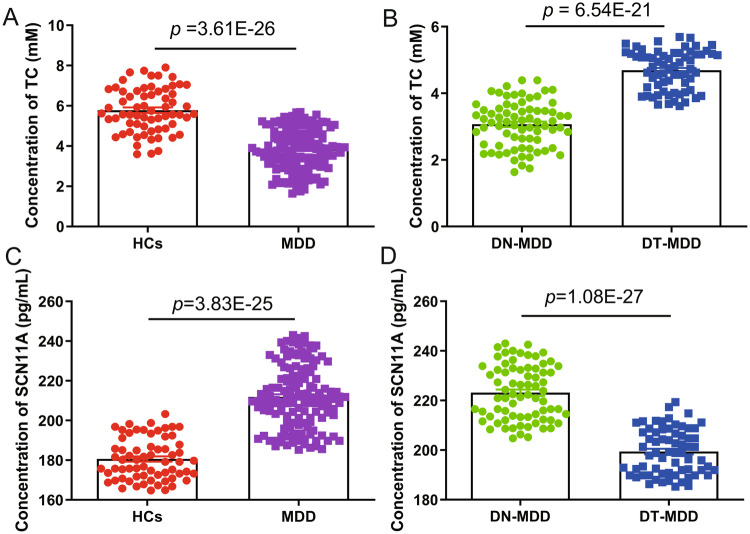
Serum levels of total cholesterol (TC) and SCN11A in major depressive disorder (MDD) patients. **A** Comparison of the serum TC levels between the groups with healthy controls (HCs) and MDD; **B** Comparison of serum TC levels between the DN-MDD and DT-MDD groups; **C** Comparison of serum SCN11A levels between the HCs and MDD groups; **D** Comparison of serum TC levels between the DN-MDD and DT-MDD groups. Data are presented as mean ± S.E.M.

Given the hypothesis that cholesterol might be correlated with MDD via SCN11A, we further detected the levels of TC and SCN11A in the serum of MDD patients. The findings showed that TC levels in MDD patients were considerably lower than in HCs (*p* = 3.61E-26; Fig. 2A), while DT-MDD patients had a considerably higher level of TC than DN-MDD patients (*p* = 6.54E-21; Fig. 2B). These results demonstrated that low lipid levels existed in MDD patients and that it was treated with antidepressant drugs to ameliorate it.

In addition, compared to HCs, MDD patients had a significantly higher level of SCN11A (*p* = 3.83E-25; Fig. 2C), whereas there was a significantly lower level of SCN11A in DT-MDD patients than in DN-MDD patients (*p* = 1.08E-27; Fig. 2D). Furthermore, compared to MDD patients with TC levels less than 2.8 mM, SCN11A levels were significantly decreased in MDD patients with TC levels more than 2.8 mM (*p* = 0.008; Fig. S1). These findings showed that SCN11A was considerably increased in MDD patients, especially in those with lower TC levels, and antidepressants might decrease the levels of SCN11A.

### Effects of different treatment modalities on TC and SCN11A levels

**Fig. 3:**
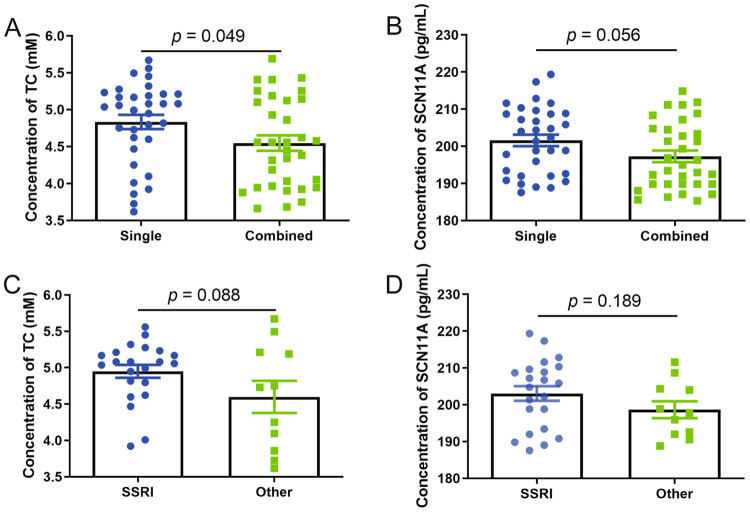
Effects of different treatment modalities on TC and SCN11A levels. **A** Effects of single antidepressant vs. combined antidepressants on TC levels; **B** effects of single antidepressant vs. combined antidepressants on SCN11A levels; **C**, **D** among the patients receiving single antidepressant, the levels of TC (**C**) and SCN11A (**D**) were similar between patients receiving selective serotonin reuptake inhibitor (SSRI) and patients receiving other antidepressants. Data are presented as mean ± S.E.M.

Table S2 shows the characteristics of antidepressants used in DT-MDD participants. Of these, 33 were given single antidepressants (22 selective serotonin reuptake inhibitors (SSRIs) and 11 others) and 33 were given combined antidepressants. In contrast to MDD patients receiving combined antidepressants, the levels of TC were significantly lower in MDD patients receiving single antidepressants (*p* = 0.049; Fig. 3A), but SCN11A did not change significantly (*p* = 0.056; Fig. 3B). Additionally, among the MDD patients receiving single antidepressant, patients receiving SSRI had the similar levels of TC (*p* = 0.088; Fig. 3C) and SCN11A (*p* = 0.189; Fig. 3D) compared to patients receiving other antidepressants.

### Correlations between SCN11A, MDD, and low lipid levels

**Fig. 4:**
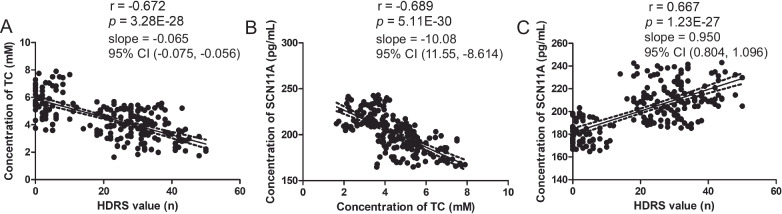
Pearson correlation between levels of TC and SCN11A and Hamilton Depression Rating Scale (HDRS) score. **A**, **B** both HDRS score (**A**) and SCN11A level (**B**) were significantly negatively correlated with TC level. **C** SCN11A level was significantly positively correlated with HDRS score.

A correlation analysis revealed a significant negative relationship between TC level and HDRS score (*r* = −0.672, *p* = 3.28E-28; Fig. 4A). Moreover, a significant negative correlation between SCN11A concentration and TC level was observed (*r* = –0.689, *p* = 5.11E-30; Fig. 4B). Meanwhile, there was a significant positive association between SCN11A level and HDRS score (*r* = 0.667, *p* = 1.23E-27; Fig. 4C). The present findings suggested that there were close relationships between SCN11A and low lipid levels in MDD patients.

### SCN11A as a diagnosing biomarker for MDD

**Fig. 5:**
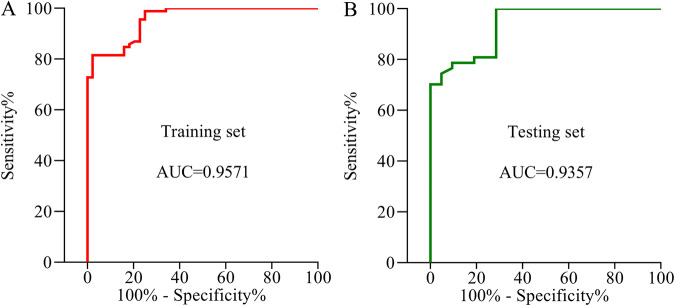
Diagnostic performance of the obtained discriminative model. **A**, **B** A discriminative model consisting of SCN11A discriminated MDD subjects from healthy controls with an area under the receiver operating characteristic curve (AUC) of 0.9571 in the training set (**A**) and 0.9357 in the testing set (**B**).

Using the training set, we obtained a discriminative model consisting of SCN11A, which could effectively separate MDD patients from HCs. The discriminative model was as follows: Y(*y* = 1) = 1/(1 + EXP(-0.206*SCN11A + 40.027)). The results of the ROC curve analysis showed that this model had an excellent diagnostic performance in diagnosing MDD patients in the training set (AUC = 0.9571; Fig. 5A). Then, the testing set was used to independently validate the diagnostic performance of this model, and the results showed that this model could still effectively diagnose MDD patients in testing set (AUC = 0.9357; Fig. 5B). These results suggested that this discriminative model could be an ‘excellent’ classifier of HCs and MDD patients, and SCN11A held the promise as a potential diagnosis biomarker for MDD.

## Discussion

In line with our earlier research, we discovered that the serum TC concentrations in MDD patients were considerably lower than in HCs. Based on the IPA database analysis, we proposed that SCN11A might serve as a link between low lipid levels and depression. Thus, this study first analyzed the serum level of SCN11A in MDD, and we found that it was considerably higher in patients with MDD than in HCs and significantly correlated with TC levels. A discriminative model consisting of SCN11A had excellent diagnostic performance in diagnosing MDD patients in both the training set and testing set. Our findings suggested that SCN11A might serve as a bridge between low lipid levels and MDD.

SCN11A, also referred to as Na_v_1.9, and originally called NaN or SNS2, is a member of the voltage-gated sodium channel family that is not susceptible to tetrodotoxin [41, 42]. It has been shown expressed within the peripheral dorsal root ganglion and throughout the central nervous system [43–46]. Studies demonstrated that Na_v_1.9 is implicated in persistent Na+ current and thus in neuronal firing rates [47, 48], and the regulation of serotonin neuron firing rates is strongly implicated in MDD [49]. A prior investigation also discovered that the sodium channel Na_v_1.9 was necessary for neurotrophin-evoked depolarization [50]. It’s interesting to note that the physiopathologic mechanism of MDD is linked to neurotrophin, such as brain-derived neurotrophic factor (BDNF) [51]. Additionally, one effective method of treating MDD is to depolarize the neurons in a targeted brain region [52]. These findings prompted us to postulate that neurotrophin might play a role in the relationship between SCN11A and the onset of MDD, which is worthy to be further investigation in the future.

Moreover, we found that the serum SCN11A level was significantly correlated to TC level (negatively) and depression severity (positively). Cholesterol is a component of lipid rafts, and it plays important functions in the viability and operation of cells [53]. Our recent study has shown that lipid metabolism in MDD patients is regulated by mesencephalic astrocyte-derived neurotrophic factor (MANF) [14]. Like BDNF, MANF is a canonical neurotrophic factor. Combined above mentioned, MANF might act as a bridge between SCN11A and MDD. Furthermore, there is strong evidence that lipid rafts play a role in the regulation of many receptors and ion channels, influencing their expression at the cell membrane as well as their activation or inactivation, open states [54–56]. The associations of cholesterol, both indirect and direct, may be responsible for these regulations. Inflammation reduces the level of cellular cholesterol, and pharmacological reduction of this substance causes nociceptive neurons to become more sensitive, promoting mechanical and thermal hyperalgesia through the stimulation of voltage-gated Na_v_1.9 channels [31]. These findings suggest that SCN11A is strongly associated with cholesterol and may be important in the pathogenesis of depression, and inflammation possibly regulates the expression of SCN11A via reduced TC levels. Meanwhile, several clinical and pre-clinical studies have demonstrated a strong association between MDD and the expression of factors that increase inflammation [57, 58], the TC and SCN11A might be the effectors of inflammation in MDD patients.

Besides, the disturbed levels of SCN11A and TC were greatly improved by antidepressant therapy. Multiple antidepressants would effectively decrease the TC levels compared to single antidepressants, while there was no significant difference in the levels of SCN11A among MDD patients who used single or multiple antidepressants, and SSRIs or other antidepressants. Paroxetine and sertraline, two effective and widely used drugs for MDD, have negative effects on the serum levels of TC and LDL-C [59, 60]. Therefore, our results could also be used as evidence to support citalopram, not paroxetine and sertraline, to be a treatment of choice for patients with depression affected by dyslipidemia [59]. Meanwhile, we postulated that the impact of antidepressants on improving SCN11A might be mediated by the regulation of cholesterol, which was deserving of further study given that SCN11A was associated with the regulation of lipids. Furthermore, considering that antidepressant drugs were commonly used in clinical practice, both DN-MDD and DT-MDD were assigned to the training and testing sets. As our previous studies conducted [32, 61], these two sets were used to evaluate the diagnostic generalizability of SCN11A. Here, we found that the SCN11A-based discriminative model had excellent diagnostic performance in diagnosing MDD patients in both the training set and testing set, which indicated that SCN11A might be a potential biomarker for diagnosing MDD.

There were few reports about the connection between MDD, low lipid levels, and SCN11A. Our findings would provide a fresh approach to looking into this relationship. Besides, it was discovered that SCN11A had been dramatically changed in the serum of MDD patients and that this alteration was associated with serum lipid and the severity of depression symptoms. This molecule may be used as an additional depressive state or characteristic biomarker. Our findings also suggested a potential function for this molecule in the development or progression of MDD, which might increase our understanding of the potential contribution of lipid metabolism to MDD. It is still unknown, though, whether depression in MDD patients is a direct result of high SCN11A levels or if it develops as a side effect. Thus, to fully understand the potential function of SCN11A in the development of MDD, longitudinal studies incorporating serum SCN11A examination are required.

This study has several limitations that need to be noted. First, patients with MDD were not compared to those with other mental conditions like schizophrenia, bipolar disorder, and so on. We need to explore if SCN11A would distinguish MDD from other psychiatric disorders that are comparable. Second, our findings suggested a close relationship between SCN11A and MDD, but could not show a causal relationship between the increased SCN11A level and MDD. Future studies should be further conducted to explore the causal connections between SCN11A level and MDD. Third, there may be a lot of protein changes in MDD, further study with large sample size confirmation is needed to determine whether the elevated levels of serum SCN11A can serve as markers for MDD. Finally, although an association between SCN11A, TC, and MDD has been found in this study, the underlying mechanisms are not known.

Taken together, we found that compared to HCs, MDD patients had significantly lower levels of TC and higher levels of SCN11A. Meanwhile, SCN11A was significantly correlated to TC concentrations and depression severity, and it could be a potential biomarker for the diagnosis of MDD. These results suggested that SCN11A might act as the bridge between low lipid levels and MDD. Our findings would be helpful for the future development of objective diagnostic methods for MDD and provide novel insights into exploring the pathogenesis of depression.

### Supplementary information

Supplemental Information

## Data Availability

The data that support the findings of this study are available from the corresponding authors upon reasonable request.
