# Supplementary material for: Elevated SCN11A concentrations associated with lower serum lipid levels in patients with major depressive disorder
Source: Transl Psychiatry. 2024 May 11;14:202. doi: 10.1038/s41398-024-02916-w (PMC11088647; doi:10.1038/s41398-024-02916-w)
Supplement: Supplementary file 1 — Supplemental Information [file 41398_2024_2916_MOESM1_ESM.pdf]

Elevated SCN11A concentrations associated with lower serum lipid levels in patients with major depressive disorder

Supplemental Information

Table S1 Characterization of the major depressive patient subjects.

Characteristics	DN-MDD (N=73)	DT-MDD (N=66)
Marital status		
Single	28 (38.36%)	21 (31.82%)
Married	40 (54.79%)	40 (60.61%)
Divorced	4 (5.48%)	4 (6.06%)
Widowed	1 (1.37%)	1 (1.52%)
Levels of education		
Low	18 (24.66%)	16 (24.24%)
Middle	21 (28.77%)	22 (33.33%)
High	34 (46.58%)	28 (42.42%)
Work situation		
Unemployed	11 (15.07%)	7 (10.61%)
Others not working	6 (8.22%)	6 (9.09%)
Working	41 (56.16%)	38 (57.58%)
In training	6 (8.22%)	4 (6.06%)
Retired	2 (2.74%)	6 (9.09%)
Housewife/-man	7 (9.59%)	5 (7.58%)
Personal history		
Drinking		
Never	57 (78.08%)	51 (77.27%)
Moderate	11 (15.07%)	7 (10.61%)
Heavy	5 (6.85%)	8 (12.12%)
Smoking		
Never	61 (83.56%)	52 (78.79%)
Moderate	10 (13.70%)	9 (13.64%)
Heavy	2 (2.74%)	5 (7.58%)
Types of antidepressants (n, %)		*Total number* = 112[#]
Fluoxetine	NA	10 (8.93%)
Sertraline	NA	8 (7.14%)
Escitalopram	NA	24 (21.43%)
Olanzapine	NA	19 (16.96%)
Mirtazapine	NA	13 (11.61%)
Venlafaxine	NA	15 (13.39%)
Others	NA	23 (20.35%)

Highest achieved educational level was determined and defined in three groups for analysis: low level of education (medium-level secondary education or below), medium level of education (higher-level secondary education or vocational

education) and high level of education (diploma level or university education).

Some patients used more than one antidepressant.

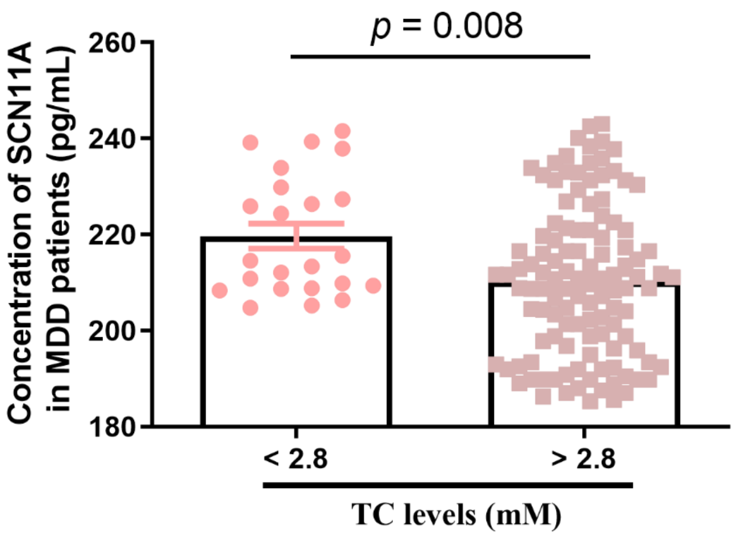

Fig S1. Comparison of the serum SCN11A levels between the MDD patients with TC levels less than 2.8 mM and MDD patients with TC levels more than 2.8 mM. Data are presented as mean ± S.E.M.

Table S2 Characteristics of the antidepressants used in DT-MDD patients.

Characteristics	Single antidepressant		Combined antidepressants	*p* value
	SSRI	Other		
Sample Size (n)	22	11	33	-
Sex (Male/Female)	10/12	5/7	12/21	0.793 [a]
Age (years)				
Range	18-59	20-65	19-66	-
Mean ± SEM	34.59±2.62	43.18±5.24	39.67±2.53	0.245 [b]
HDRS (Mean ± SEM)	28.09±1.69	23.82±1.20	28.30±1.86	0.052 [b]
Duration of illness (months)				
Range	2-180	8-132	1-144	-
Mean ± SEM	47.05±9.51	64.00±13.42	54.79±8.39	0.527 [b]

Continuous variables are expressed as Mean ± Standard Error of the Mean (SEM).
DT-MDD, drug-treatment major depressive disorder; *HDRS*, hamilton depression rating scale; *HCs*, healthy controls. *SSRI*, selective serotonin reuptake inhibitors.
[a] Analyzed by the Chi-square test; [b] Analyzed by Kruskal-Wallis H test.
